# Latent class analysis of violence against adolescents and psychosocial outcomes in refugee settings in Uganda and Rwanda

**DOI:** 10.1017/gmh.2017.17

**Published:** 2017-10-16

**Authors:** S. R. Meyer, G. Yu, S. Hermosilla, L. Stark

**Affiliations:** 1 Columbia University Mailman School of Public Health, Population and Family Health, New York, NY, USA; 2 New York University, Rory Meyers College of Nursing, New York, NY, USA; 3 Department of Psychiatry, Columbia University, New York, USA

**Keywords:** Refugees, adolescents, violence, displacement, depression, anxiety

## Abstract

**Background:**

Little is known about violence against children in refugee camps and settlements, and the evidence-base concerning mental health outcomes of youth in refugee settings in low and middle-income countries is similarly small. Evidence is needed to understand patterns of violence against children in refugee camps, and associations with adverse mental health outcomes.

**Methods:**

Surveys were conducted with adolescent refugees (aged 13–17) in two refugee contexts – Kiziba Camp, Rwanda (*n* = 129) (refugees from Democratic Republic of Congo) and Adjumani and Kiryandongo refugee settlements, Uganda (*n* = 471) (refugees from South Sudan). Latent Class Analysis was utilized to identify classes of violence exposure (including exposure to witnessing household violence, verbal abuse, physical violence and sexual violence). Logistic regressions explored the association between latent class of violence exposure and symptoms of depression and anxiety.

**Results:**

In Rwanda, a two-class solution was identified, with Class 1 (*n* = 33) representing high levels of exposure to violence and Class 2 (*n* = 96) representing low levels of exposure. In Uganda, a three-class solution was identified: Class 1 (high violence; *n* = 53), Class 2 (low violence, *n* = 100) and Class 3 (no violence, *n* = 317). Logistic regression analyses indicated that latent violence class was associated with increased odds of high anxiety symptoms in Rwanda (AOR 3.56, 95% CI 1.16–0.95), and high *v*. no violence class was associated with depression (AOR 3.97, 95% CI 1.07–7.61) and anxiety symptoms (AOR 2.04, 95% CI 1.05–3.96) in Uganda.

**Conclusions:**

The present results support the existing evidence-base concerning the association between violence and adverse mental health outcomes, while identifying differences in patterns and associations between refugee youth in two different contexts.

## Introduction

Violence against children is increasingly recognized as an urgent public health concern, with prevalence studies indicating high levels of exposure to violence globally, and strong associations of exposure to violence with adverse physical, mental and behavioral health outcomes (Hillis *et al*. 2016; Sumner *et al*. 2016). Long-term impacts of exposure to violence in childhood include substance and alcohol-use, chronic diseases, sexual risk behaviors and poor self-rated health and life satisfaction (Felitti *et al*. 1998; Norman *et al*. 2012; Mersky *et al*. 2013). A systematic review of child maltreatment (including physical abuse, emotional abuse and neglect) found strong associations with subsequent risk of depressive and anxiety disorders (Norman *et al*. 2012).

Violence against children and adverse psychosocial outcomes are of particular concern in refugee settings. Various ecological, household and individual risk factors that increase violence against children operate in such contexts (Rubenstein & Stark, 2017). Conflict-induced displacement can result in disrupted social structures, fragmented child protection systems and increased risks for perpetration of violence against children, including loss of caregivers’ livelihoods and lack of access to basic needs (Reed *et al*. 2011; Cardoso *et al*. 2016).

In studies of mental health of refugee children, exposure to violence is an established risk factor for adverse psychosocial outcomes. For example, a systematic review of risk and protective factors for children affected by conflict found that ‘[e]xposure to violence is the factor with the strongest evidence base for the risk of subsequent psychological disturbances,’ noting that evidence has shown that ‘[t]he degree of direct exposure to threat, cumulative number of adverse events, and duration of exposure, all consistently increased the odds of mental health symptoms’ (Reed *et al*. 2011). A systematic review of risk and protective factors for child soldiers identified a number of studies that found associations between exposure to violence amongst child soldiers, and mental health and psychosocial problems (Betancourt *et al*. 2013). Taken as a whole, these reviews indicate a strong correlation between exposure to violence and adverse mental health outcomes in humanitarian contexts.

Within the existing evidence base on violence and psychosocial outcomes, the vast majority of research focuses on refugee children who have been resettled to high-income settings; few studies have focused on refugee youth located in rural refugee camps or settlements, in low and middle-income countries (LMIC). While there are many overlapping risk and protective factors for mental health outcomes for refugee children in LMIC and in high-income settings, there are important contextual differences between refugee camps in LMIC and resettlement settings in high-income countries that may influence psychosocial outcomes. A systematic review of mental health of displaced children in high-income settings identified several factors influencing mental health that are less relevant or not present in refugee camps, for example, racial discrimination and socio-cultural adaptation (Fazel *et al*. 2012), whereas refugee camps in LMIC are often characterized by on-going threats to security and well-being, with often limited access to meet refugees’ basic needs (Reed *et al*. 2011). An improved understanding of adversities faced by refugee youth living in camps and settlements, and associations with health outcomes, is needed (Reed *et al*. 2011). In addition, the needs of adolescents affected by conflict and displacement, which vary by sex and context, have often been overlooked in child protection and child health programming for refugees (Plan International, 2016). Adolescence is a period of transition with important physical, social and cognitive changes, including development of decision-making skills and positive sense of self and self-efficacy (Blum *et al*. 2014); further evidence concerning the impacts of violence on adolescent refugees is needed to inform policy and program response.

Methodological approaches to understanding the associations between exposure to violence and subsequent psychosocial outcomes have advanced to account for recognized patterns in violence victimization. Latent class analysis (LCA) is an approach that uncovers underlying ‘classes’ of exposures in order to better understand patterns of exposures and associations to outcomes (Yu *et al*. 2015), for example, patterns of exposure to childhood maltreatment and subsequent violence perpetration (Davis *et al*. 2015) and childhood maltreatment profiles and subsequent clinical diagnosis of mental disorders (Witt *et al*. 2016). LCA has been used to identify patterns of exposure to violence, and identify heterogeneous groups of women or children, for example, taking into account variations in types of exposure and identifying sub-groups within a sample to further explore dynamics of violence and associations with health outcomes (Davies *et al*. 2015). These approaches have been used to a much more limited degree amongst children and adolescents (Nooner *et al*. 2010; McChesney *et al*. 2015; Choi *et al*. 2016). LCA has been utilized to an even lesser degree in low and middle-income settings (Clarke *et al*. 2016; Ismayilova *et al*. 2016), and in humanitarian contexts (Sipsma *et al*. 2015). These studies have found significant contrasts to LCA studies focused on maltreatment and child abuse in high-income settings, and further analysis of patterns of violence against children in humanitarian contexts is warranted.

In this study, we aim to expand the evidence-base examining exposure to violence and patterns of violence by using LCA, as well as exploring associations with psychosocial outcomes. The analysis, following the analytic framework guiding previous work on adverse childhood events (ACEs) (Anda *et al*. 2006), recognizes the potentially deleterious effect of various types of direct and indirect exposure to violence, given strong dose-response associations seen between all types of ACEs and physical and mental health outcomes. As Mersky *et al*. summarize, ‘similar consequences can result from different antecedent risks’ (Mersky *et al*. 2013), and direct exposure to physical and sexual violence, as well as witnessing violence in the household, are included in this study to reflect evidence (Bair-Merritt *et al*. 2006) and this conceptual approach.

The study focuses on adolescent refugees in two refugee contexts – refugees from Democratic Republic of Congo (DRC) living in Kiziba Camp in Rwanda, and refugees from South Sudan living in Kiryandongo and Adjumani refugee settlements in Uganda. We ask the following research questions: (1) Are there distinct latent classes of violence exposure, (2) Do these latent classes differ for the two populations (refugees from DRC in Rwanda, and refugees from South Sudan in Uganda), (3) How do these classes differ by socio-demographic variables, and (4) Do these distinct latent classes of violence exposure have differential associations with symptoms of depression and anxiety?

## Methods

### Study setting and sample

The current study draws from the surveys conducted in Kiziba Camp, Rwanda (2013) and Kiryandongo and Adjumani refugee settlements, Uganda (2014–2015), as part of the ‘Measuring Impact though a Child Protection Index’ study.

Kiziba Camp was established in December 1996. Refugees in Kiziba Camp fled the DRC due to armed conflict. Violence in DRC between 1996 and 1998 generated large-scale population movement from DRC to Rwanda. On-going violence and insecurity in eastern DRC has blocked repatriation of refugees from DRC in Rwanda, and the situation of refugees from DRC in Rwanda is considered protracted, lacking immediate durable solutions.

Data collection was conducted in Kiryandongo and Adjumani settlements, Uganda. In December 2014 (Kiryandongo) and March 2015 (Adjumani). Outbreaks of violence occurred across South Sudan in December of 2013; as the conflict grew in intensity and complexity, civilians from several different regions were forced to flee to neighboring countries, including Northern Uganda.

Systematic random sampling was used in both countries. In Kiziba Camp, a complete sampling frame of quartiers and households was available. A total of 129 households were included in the survey in Rwanda. In Kiryandongo and Adjumani refugee settlements, given the changing nature of the refugee population due to active influx of new refugees from South Sudan, the sampling frames available from international agencies did not accurately reflect household numbers and population size. The research team conducted a mapping exercise to supplement the limited existing data on households. In both settings, final households were selected using a sampling interval based on the number of households, target sample size, expected prevalence of households with eligible adolescent respondents and expected refusal rate. In each household, data collectors listed the number of eligible adolescents, and randomly selected an adolescent for participation, to reduce selection bias (for example, only including adolescents at home while a data collector visited). A total of 251 households were included in Adjumani and 220 were included in Kiryandongo, for a total sample of 471 in Uganda.

### Procedures

Data collector teams in each country received 8 days of training focusing on interviewing children about violence and human subjects research procedures. The survey instruments were developed in English and translated into appropriate languages (for Rwanda, Kinyarwanda, and for Uganda, Dinka and Nuer), with group checking of translations during data collector training. In Rwanda, the data were collected using paper surveys, and inputted into EpiInfo, with 10% of surveys double-checked to ensure accurate data entry. In Uganda, all surveys were administered using smap, a mobile phone-based survey program.

### Measures

#### Socio-demographic characteristics

Survey instruments asked adolescents to report several demographic characteristics, including school attendance, educational attainment, and living status with regard to their parent or caregiver.

#### Violence exposure

Adolescents’ exposure to violence and abuse was assessed using questions adapted from studies of Violence against Children designed by the Centers for Diseases Control and the ISPCAN Child Abuse Screening Tool – Children's Version (The Ministry of Gender, 2013; ISPCAN, 2015). Table 1 indicates the questions asked, encompassing witnessing household violence, and experiencing verbal abuse, physical violence and sexual violence. A total of 14 separate items assessing exposure to any type of violence were included in surveys in both countries, and are included in the present analysis. All items in the survey assessed both lifetime prevalence and past-year prevalence. Lifetime prevalence reports are utilized in the present analysis, given survey questions focused on lifetime experience (ever having experienced) are less susceptible to telescoping bias than past-year questions.

**Table 1.** Violence questions included in survey

Type of violence	Questions
Witnessing violence	*‘Has anyone in your home ever used drugs and/or alcohol and then behaved in a way that frightened you?’, ‘Have you ever seen adults in your home shouting and yelling at each other* (*arguing*) *in a way that frightened you?’, ‘Have you seen adults in your home hit, kick, slap, punch each other or hurt each other physically in other ways?’* and, ‘*Have you ever seen anyone in your home use knives, guns, sticks, rocks or other things to hurt or scare someone else inside the home?’*
Verbal abuse	*‘Has anyone in your family or living in your home ever screamed at you very loudly and aggressively?’, ‘Has anyone in your family or living in your home ever called you names, said mean things or cursed you?’, ‘Has anyone in your family or living in your home ever said that they wished you were dead/ had never been born?’, ‘Has anyone in your family or living in your home ever threatened to leave you forever or abandon you?’,* and ‘*Has anyone in your family or living in your home ever threatened to hurt or kill you, including invoking evil spirits against you?’*
Physical violence	*‘Has anyone in your family or living in your home ever pushed, grabbed or kicked you?’, ‘Has anyone in your family or living in your home ever hit, beat or spanked you with a hand?’* and ‘*Has anyone in your family or living in your home ever threatened you with a knife or a gun?’*
Sexual violence	*‘Was there a time when you were physically forced to have sexual intercourse against your will?’* and ‘*Was there a time when you were persuaded or pressured to have sexual intercourse against your will?’*

#### Psychosocial outcomes

The psychosocial outcomes were symptoms of anxiety and depression. The analysis of psychosocial outcomes entailed generating a binary variable of high *v*. low symptom levels, given the outcome data did not meet requirements for ordinary least squares regression. Analysis of the binary variable does not indicate a clinical cut-off, for example, depressed *v*. not depressed, but rather indicates higher *v*. lower levels of symptoms. Sensitivity analyses were conducted, with multiple logistic regressions of psychosocial outcome measures, utilizing a cut-off one point below and one-point above the cut-offs used in this analysis, and these analyses indicated findings consistent with the results using the cut-off points utilized in this analysis.

##### Anxiety

Levels of anxiety among adolescent participants were assessed using the 5-item version of the Screen for Child Anxiety Related Disorders (SCARED), assessing anxiety symptoms experienced in the past 3 months, with a 3-point scale (range 0–10) (Birmaher *et al*. 1999). Means were imputed for missing values of those participants who refused one question of the scale (Uganda *n* = 6, 1.26%; Rwanda *n* = 2, 1.56%); no participants refused more than one item. The Cronbach's alpha for this scale in Uganda was 0.70 and in Rwanda was 0.73. Scale scores were transformed into a binary score of ‘high’ levels of symptoms of anxiety (*v*. not high) using a cutoff score of four and above as ‘high’ (Kohrt *et al*. 2008).

##### Depression

Different depression outcome measures were selected for each country, based on findings from instrument pilot testing, translation of instruments and input from data collectors. In Uganda, symptoms of depression within the past 2 weeks among adolescent participants were assessed using the Mood and Feelings Questionnaire Child Self Report (MFQ-C), short version (Angold & Costello, 1987). The scale consists of 13 questions scored on a 3-point scale, with three response options: true, sometimes true and not true. Means were imputed for missing values of those participants who refused three or fewer questions of the scale; no participants refused more than three. The Cronbach's alpha for this scale was 0.79. We transformed the continuous outcome into a binary variable using 12 and above as the ‘high symptoms’ group, to ensure a similar distribution of high *v*. low symptoms to the Rwanda depression outcome measure.

In Rwanda, depressive symptoms were assessed using the emotional symptoms sub-scale of the Strengths and Difficulties Questionnaire (SDQ). The SDQ assesses non-clinical psychological distress, and has been used in a number of humanitarian settings with child and adolescent populations (Panter-Brick *et al*. 2011; Tol *et al*. 2012). The emotional symptoms sub-scale consists of five questions on a 3-point scale, with three response options: not true, somewhat true, and certainly true. Scale scores were transformed into a binary score of 5 and above as the cut-off indicating high levels of symptoms. The Cronbach's alpha for the sub-scale was 0.59.

### Analysis

LCA was used to identify classes of individuals reporting similar patterns of exposure to violence. Maximum likelihood methods were used to group individuals into distinct ‘classes’ of violence exposure, with LCA conducted separately for the Uganda sample and the Rwanda sample. Dichotomous violence exposure indicators were modeled with a binomial logit link and the overall count of violence exposures was modeled with a log Poisson link. Bayesian Information Criterion (BIC) was used to determine the optimal number of classes per sample; BIC balances model fit and parsimony. LCA analysis was conducted using Mplus version 6.11 (Muthen & Muthen, 1998–2011). Online Supplementary File 1 shows the fit statistics for each sample. The optimal solution for Uganda is the three-class solution based on the BIC, the Vuong-Lo-Mendell-Rubin likelihood ratio (VLMR LR) and the Lo-Mendell-Rubin Adjusted LRT test comparing the three-class solution with the four-class solution. For Rwanda, the optimal solution for Rwanda is the two-class solution based on the BIC, the VLMR LR and the Lo-Mendell-Rubin Adjusted LRT test comparing the two-class solution with the three-class solution. After determining the optimal number of classes, Bayes’ Rule was used to determine the posterior probability that an individual belonged to a certain class.

Having determined the number of classes per country sample, the following socio-demographic variables were reported, for the overall country sample and by violence class – gender, age, length of time in camp, parental living status (no living biological parents, one living biological parent and both biological parents living), level of education and household size, as well as calculating prevalence of high and low symptoms of psychosocial outcomes, overall and by violence-class. Chi-squared tests were utilized to determine differences in categorical demographic variables and psychosocial outcomes between violence classes, Student's *t* test was used for normally distributed continuous predictors, and Wilcoxon rank sum test and Kruskal–Wallis test were used for non-normally distributed continuous predictors. Finally, multiple logistic regression models were run separately for each psychosocial outcome, using violence class as the primary exposure, and including age, gender, parental status, household size and education as potential confounders. Logistic regression analysis was conducted using Stata 14.

### Ethical approval

The research was covered under the Columbia University Medical Center IRB AAAB7134. In Uganda the Office of the Prime Minister, granted ethical clearance while in Rwanda the UNHCR and AVSI Rwanda reviewed ethical protocol in accordance with existing Rwandan camp-based research regulations.

## Results

Table 2 reports the results of the LCA of violence exposure. In Rwanda, a two-class solution was identified, with Class 1 representing high levels of exposure to violence and Class 2 representing low levels of exposure. In Rwanda, for Class 1, there were seven violence items that had more than a 10% prevalence increase from the overall average. For Class 2, there were no violence items that had a more than 10% prevalence increase from the overall average. Using this two-class model, 26% of the sample (*n* = 33) was determined to be in Class 1 and 74% of the sample (*n* = 96) was determined to be in Class 2. In the full sample in Rwanda, respondents reported having experienced an average of 2.2 exposures to violence (out of a total of 14 items), whereas in the high-violence class mean exposure was 5.4 and in the low-violence class mean exposure was 1.1. In terms of types of exposures, the high-violence Class 1 respondents reported exposure to witnessing violence (for example, 90.9% reported having witnessed adults home shouting and yelling at each other (arguing) in the household in a way that frightened them), verbal abuse (21.4% reported having had someone in their family or home threaten to leave them), physical assault (64.8% reported having been hit, beaten or spanked) and sexual violence (15.0% reported having been coerced to have sex against their will). The Class 2 low-violence exposure respondents primarily reported exposure to witnessing violence in the household, for example, 65.5% reported having seen adults shouting and yelling in a way that frightened them in the home, and 12.8% reported having seen adults hit, kick, slap, punch each other or hurt each other physically in the home.

**Table 2.** Latent class analysis of violence exposure, Rwanda and Uganda

	Rwanda			Uganda			
	Average	Class 1 high violence	Class 2 low violence	Average	Class 1 high violence	Class 2 low violence	Class 3 no violence
Violence items							
Witnessing drug and alcohol use	12.4%	19.0%	10.1%	8.5%	29.1%	24.1%	0%
Adults arguing	72.1%	90.9%	65.5%	14.0%	50.4%	38.4%	0%
Adults assaulting	17.8%	32.1%	12.8%	10.9%	51.6%	22.8%	0%
Weapons use	3.1%	12.0%	0.0%	4.9%	24.3%	9.7%	0%
Screamed at child	24.8%	76.2%	6.9%	11.7%	58.0%	23.3%	0%
Name-calling at child	9.3%	30.0%	2.1%	6.8%	46.8%	6.5%	0%
Wish bad things at child	4.7%	12.0%	2.1%	4.5%	30.5%	4.4%	0%
Threatened to leave child	6.2%	21.4%	0.9%	9.8%	62.3%	12.1%	0%
Threatened to hurt child	1.6%	6.0%	0.0%	5.7%	49.6%	0.0%	0%
Pushed, grabbed, kicked child	10.9%	37.8%	1.5%	11.1%	77.1%	10.0%	0%
Hit, beaten or spanked with a hand	18.6%	64.3%	2.7%	13.8%	76.8%	23.1%	0%
Threatened with knife	1.6%	6.0%	0.0%	4.7%	33.0%	4.0%	0%
Sexually coerced against will	3.9%	15.0%	0.0%	3.2%	16.4%	6.0%	0%
Pressured to have sex	1.6%	6.0%	0.0%	1.5%	9.2%	2.0%	0%
Average number of violence items experienced	2.2	5.4	1.1	1.1	6.2	1.9	0
*N*	129	33	96	470	53	100	317
%	100%	26%	74%	100%	11%	21%	68%

Grey highlights designate violence items that have more than a 10% prevalence increase from the average, overall.

The percent in each class reporting is the posterior probability prevalence, by sample.

In Uganda, a three-class solution was identified: Class 1 (high violence), Class 2 (low violence), and Class 3 (no violence). In Uganda, in Class 1, there were 13 items that had more than a 10% prevalence increase from the overall average; for Class 2, five items, and for Class 3, no items. Using this three-class model, 11% of the sample (*n* = 53) was determined to be in Class 1, 21% of the sample (*n* = 100) was determined to be in Class 2, and 68% of the sample (*n* = 317) was determined to be in Class 3. The mean exposure to violence across the full sample was 1.1; respondents in Class 1 were exposed to a mean of 6.2 exposures, respondents in Class 2 were exposed to a mean number of 1.9 exposures, and Class 3 respondents reported no exposure to violence. Respondents in Class 1, high-violence, reported exposures across witnessing violence, verbal abuse, physical assault and sexual violence, whereas Class 2 respondents primarily reported witnessing violence in the household and physical assault, with some reporting of exposure to verbal abuse.

Table 3 displays socio-demographic variables and psychosocial outcomes, for the full sample per country, and by violence-class. There were no differences in socio-demographic variables across violence classes; that is, gender, age, length of time in refugee context, parental living status, level of education and household size did not significantly predict membership in latent violence class. In Rwanda, some differences in prevalence of psychosocial outcomes were found by violence class, with a significant difference in proportion of respondents in the Class 1 high-violence class reporting high levels of anxiety compared with the Class 2 low-violence class (high levels of symptoms: Class 1: 24.4% *v*. Class 2: 8.33%, *p* = 0.017). In Uganda, depression symptoms and anxiety symptoms differed significantly by latent violence class. For depression symptoms, 50.94% of respondents in Class 1 high-violence reported high levels of symptoms of depression, compared with 36.00% in Class 2 and 17.98% in Class 3 (*p* < 0.001). For symptoms of anxiety, 37.74% of respondents in Class 1 reported high levels of symptoms of anxiety, compared with 23.00% in Class 2 and 19.56% in Class 3 (*p* = 0.013).

**Table 3.** Socio-demographics

	Rwanda *N* = 129 *N* (%)	High violence class	Low violence class	*X*^2^(df)	*p* value	Uganda *N* = 470 *N* (%)	High violence class	Low violence class	No violence class	*X*^2^(df)	*p* value
Gender											
Male	52 (40.31%)	16 (48.48%)	36 (37.50%)	1.23 (1)	0.267	246 (52.34%)	27 (50.94%)	60 (60.00%)	159 (50.16%)	3.00 (2)	0.223
Female	77 (59.69%)	17 (51.52%)	60 (62.50%)			224 (47.66%)	26 (49.06%)	40 (40.00%)	158 (49.84%)		
Age											
13–15	61 (47.29%)	16 (48.48%)	45 (46.88%)	0.03 (1)	0.873	331 (70.43%)	36 (67.92%)	64 (64.00%)	231 (72.87%)	3.05 (2)	0.217
16–17	68 (52.71%)	17 (51.52%)	51 (53.13%)			139 (29.57%)	17 (32.08%)	36 (36.00%)	86 (27.13%)		
Length of time in Rwanda/Uganda (months)	Median (IQR) 14.5 (11–17)	13 (11–17)	15 (12–17)		0.3173	Median (IQR) 12 (9–12)	11 (7–12)	11 (9–12)	12 (10–12)	5.01 (2)	0.0819
Parental living status											
No living biological parents	9 (6.98%)	1 (3.03%)	8 (8.33%)	1.98 (2)	0.371	38 (8.15%)	7 (13.46%)	6 (6.06%)	25 (7.94%)	9.30 (4)	0.054
One living biological parent	34 (26.36%)	7 (21.21%)	27 (28.13%)			174 (37.34%)	26 (50.00%)	32 (32.32%)	116 (36.83%)		
Both biological parents living	86 (66.67%)	25 (75.76%)	61 (63.54%)			254 (54.51%)	19 (36.54%)	61 (61.62%)	174 (55.24%)		
Level of education											
Never attended school	0 (0.00%)	0 (0.00%)	0 (0.00%)	0.29 (1)	0.588	356 (75.74%)	38 (71.70%)	75 (75.00%)	243 (76.66%)	5.18 (4)	0.269
Primary or below	81 (64.80%)	22 (68.75%)	59 (63.44%)			87 (18.51%)	10 (18.87%)	16 (16.00%)	61 (19.24%)		
Some secondary or above	44 (35.20%)	10 (31.25%)	34 (36.56%)	*t* (df)		27 (5.74%)	5 (9.43%)	9 (9.00%)	13 (4.10%)		
Household size	Mean (s.d.) 7.11 (2.64)	7.39 (0.43)	7.01 (0.28)	−0.72 (126)	0.4738	Median (IQR) 7 (6–9)	7 (6–10)	8 (6–10)	7 (6–9)	4.411 (2)	0.1102
Psychosocial outcomes				*X*^2^ (df)							
Emotional symptoms/depression	SDQ sub-scale					MFQ					
<5	98 (75.97%)	24 (72.73%)	74 (77.08%)	0.26 (1)	0.613	<12 350 (74.47%)	26 (49.06%)	64 (64.00%)	260 (82.02%)	33.27 (2)	<0.001
≥5	31 (24.03%)	9 (27.27%)	22 (22.92%)			≥12 120 (25.53%)	27 (50.94%)	36 (36.00%)	57 (17.98%)		
Anxiety SCARED	Mean (s.d.)					Mean (s.d.)					
<4	113 (87.60%)	25 (75.76%)	88 (91.67%)	5.72 (1)	0.017	365 (77.66%)	33 (62.26%)	77 (77.00%)	255 (80.44%)	8.68 (2)	0.013
≥4	16 (12.40%)	8 (24.24%)	8 (8.33%)			105 (22.34%)	20 (37.74%)	23 (23.00%)	62 (19.56%)		

Table 4 displays results of the multiple logistic regression models, exploring the association of latent violence class membership and psychosocial outcomes. In Rwanda, with low-violence class as the reference category, membership in the high-violence class was significantly associated with increased odds of high symptoms of anxiety (AOR 3.56, 95% CI 1.16–10.95). In Uganda, with no-violence class as the reference category, membership in low-violence *v*. no-violence class increased the odds of high symptoms of depression (AOR 3.13, 95% CI 1.83–5.36), but was not significantly associated with anxiety symptoms. Membership in high-violence class *v*. no-violence class was significantly associated with higher levels of symptoms of depression (AOR 3.97, 95% CI 1.07–7.61) and higher levels of symptoms of anxiety (AOR 2.04, 95% CI 1.05–3.96).

**Table 4.** Latent classes and psychosocial outcomes

	Rwanda		Uganda	
	Depression/ emotional symptoms	Anxiety	Depression/ emotional symptoms	Anxiety
	High *v*. low	High *v*. low	High *v*. low	High *v*. low
Violence class				
None	N/A	N/A	REF	REF
Low	REF	REF	AOR 3.13*** (1.83–5.36)	AOR 1.36 (0.77–2.42)
High	AOR 1.43 (0.54–3.79)	AOR 3.56* (1.16–10.95)	AOR 3.97*** (1.07–7.61)	AOR 2.04* (1.05–3.96)
Covariates				
Age	AOR 1.57** (1.15–2.14)	AOR 1.36 (0.93–1.98)	AOR 0.97 (0.83–1.14)	AOR 1.00 (0.85–1.18)
Gender	AOR 1.12 (0.46–2.71)	AOR 1.45 (0.46–4.59)	AOR 1.48 (0.94–2.34)	AOR 2.27** (1.41–3.66)
Parental status	AOR 0.54 (0.20–1.42)	AOR 1.80 (0.47–6.89)	AOR 0.27*** (0.17–0.43)	AOR 0.35*** (0.22–0.57)
Household size	AOR 1.12 (0.94–1.33)	AOR 0.89 (0.00–0.17)	AOR 0.98 (0.92–1.04)	AOR 1.04 (0.98–1.10)
Ever education	–^a^	–^a^	AOR 0.74 (0.44–1.24)	AOR 0.49** (0.30–0.82)

*p < 0.05, **p < 0.01, ***p < 0.001.

a For Rwanda, no adolescents reported never attended school.

Additionally, certain socio-demographic variables were significantly associated with psychosocial outcomes. In Uganda, parental status was significant for both depression and anxiety symptoms; having both parents alive *v*. having either one or none alive was associated with lower symptoms of depression (AOR 0.27, 95% CI 0.17–0.43) and anxiety (0.35, 95% CI 0.22–0.57) In Uganda, being female was significantly associated with higher levels of symptoms of anxiety (AOR 2.27, 95% CI 1.41–3.66).

## Discussion

The comparison of datasets from two different refugee contexts – Kiziba Camp, a protracted refugee setting in Rwanda, with adolescent refugees from DRC, and two refugee settlements in Uganda, in the midst of emergency influx from South Sudan – provides evidence, in regards to our research questions, that the latent classes differ for the two populations, and that there are differential associations between latent class and mental health outcomes.

The primary exposures driving the difference in class solution was prevalence of exposure to witnessing violence in the household, with results indicating significantly higher levels of witnessing of household violence in Kiziba Camp compared with the refugee settlements in Uganda. The long-term nature of displacement in Kiziba Camp, where many refugees have lived more than 20 years, may have contributed to higher levels of household violence, with protracted displacement considered a risk factor for alcohol-use and mental disorders, which may raise risk of intimate partner violence (Feseha *et al*. 2012; Hossain *et al*. 2014). This finding confirms evidence that indicates that household violence, including violence against children and intimate partner violence, is of particular concern in conflict-affected settings (Stark & Ager, 2011), yet it is unclear why witnessing of levels of household violence may be lower at the time of research in refugee settlements in Uganda. An ecological framework has been suggested to improve understanding of the associations between humanitarian emergencies and violence against children; in outlining this framework, Rubenstein and Stark indicate that the ‘relationship between humanitarian emergencies and violence against children will differ across contexts due to variability in the underlying population characteristics, gender roles and features of the emergency itself’ (Rubenstein & Stark, 2017), as well as host country policies and camp/settlement conditions. Host country policies may play a role in influencing patterns of violence in these two contexts: refugee-hosting policies in Rwanda and Uganda differ, with refugees in Rwanda confined to camps that are often remote and lacking in livelihood opportunities (as is the case for Kiziba Camp), whereas Uganda's refugee hosting policy allows increased integration and interaction with local host communities (Stark *et al*. 2015). However, levels of household violence are influenced by a multiplicity of factors, and further study could help to elucidate the factors relating to differences in patterns of violence identified in this study, which may in turn be able to inform contextually-based and targeted policy and programming recommendations to reduce violence exposure.

Similarly, the differential associations between violence exposure patterns and mental health outcomes between the populations may indicate different patterns of expression of distress and symptomology between Congolese and South Sudanese adolescents, yet further data are needed to understand these potentially distinct expressions of mental health and well-being. The majority of existing evidence on Congolese adolescents affected by conflict is focused on adolescents still residing in DRC, and has focused on coping strategies and resilience (Cherewick *et al*. 2015), sexual violence, stigma and mental health (Verelst *et al*. 2014*a*, *b*), risk factors for physical, emotional and sexual violence (Stark *et al*. 2017) and the association of caregivers’ gender attitudes and acceptance of IPV and violence exposure (Falb *et al*. 2017). Research on refugees from South Sudan is even more sparse, with some research focusing on the dose–response relationship between traumatic events and post-traumatic stress disorder amongst adult South Sudanese refugees in Uganda (Neuner *et al*. 2004) and priority mental health problems and resources amongst adult South Sudanese refugees in Uganda (Adaku *et al*. 2016), with some qualitative data indicating that ‘thinking too much’ is an important form of distress and framing of symptoms amongst South Sudanese (Coker, 2004; Goodman, 2004). Taken as a whole, this evidence-base supports the findings in the present study, indicating high levels of exposure to violence and associations with mental health outcomes, however, does not shed light on the particular patterns of violence identified in this study, or on the associations with symptoms of anxiety and not with depression symptoms. Moreover, given this study compares two different host countries *and* populations from two different countries of origin, it is not possible to disentangle the influences of prior or current cultural, social and structural factors on mental health outcomes. However, the findings indicate points of departure for program planning and policy response, as well as future research directions.

Compared with other LCA analyses exploring patterns of violence, this study identified a smaller number of latent classes, likely given that many other LCA analyses focus specifically on abused and maltreated populations utilizing protective services (Witt *et al*. 2016), whereas this is a community sample, which includes respondents who did not disclose any experience of abuse. In addition, this analysis identified classes that are primarily interpretable in terms of level of exposure, rather than type of exposure, in comparison with other studies of child maltreatment or exposure to intimate partner violence (McChesney *et al*. 2015; Clarke *et al*. 2016). Despite these differences, the associations of severity of exposure (in the case of this study, high *v*. low, or high *v*. low *v*. no) and adverse mental health outcomes is consistent with several LCA studies (Davies *et al*. 2015), as well as data indicating a dose–response relationship between exposure to ACEs and poor health outcomes (Anda *et al*. 2006).

Findings regarding the associations between violence and mental health outcomes confirm data on refugees resettled in high-income settings, however, existing data do not allow for a detailed comparison of risk and protective factors between refugees in LMIC and in high-income contexts. While refugee children in high-income settings face uncertainty relating to asylum procedures, refugee children in protracted refugee settings in LMIC are often caught between having no concrete opportunities for legal integration in their host country, and no apparent possibility for return to their home country (Loescher, 2011). While distinct differences were not identified in this study compared with the body of existing literature on refugee children resettled to high-income settings, the differences in context are substantial enough to warrant considerations for future comparative analysis.

Implications for policy and programming from this study include aspects of prevention of and response to violence. In prevention programming, emerging evidence concerning effectiveness of parenting programs and community-based programs to change gender inequitable norms (Lundgren & Amin, 2015) should be rigorously tested for effectiveness in refugee contexts. In terms of response to exposure to violence, improvements in services to address mental health problems of adolescents in refugee camps and settlements are needed. Utilization of health services for mental and neurological disorders in refugee camps is low for emotional disorders, including anxiety and depression, and health care services for emotional disorders may not address population needs in refugee contexts (Kane *et al*. 2014). Services must be designed and delivered with accessibility and appropriateness for adolescents in mind, including, for example, entry points to services in multiple spaces within refugee camps, support for utilization of services from peers, mentors, teachers and caregivers, and efforts to reduce stigma associated with utilizing mental health services. These findings also indicate that programming and policies will not operate uniformly across contexts and populations, and understanding the pathways between violence and well-being is critical for developing and targeting appropriate interventions. Packages of evidence-based interventions to prevent and respond to violence against children – for example, the new World Health Organization INSPIRE package (WHO, 2016) – provide the basis for intervention development, but lack clarity on what conditions may make certain interventions effective.

### Limitations

The results should be interpreted in light of several limitations. We did not assess for severity, duration or perpetrator of violence, and therefore this data could not be included in order to further parse the classes of violence exposure. Some of the confidence intervals are wide, due to small cell counts in some violence classes. The associations identified are taken from data from one time point, and therefore causality between violence exposure and psychosocial outcomes cannot be definitively assessed. However, psychosocial scales inquired about symptoms from a relatively short time period prior to the survey (a maximum of 6 months), and it is plausible to assume that the reported exposures to violence preceded reported symptoms. Differences in the two versions of the mental health outcome measures in Uganda (Nuer and Dinka languages), and differences in the depression measure utilized between Rwanda and Uganda may have introduced measurement error. The present LCA focused solely on violence; other studies have included other adversities within latent classes, and expansion of the types of adversities experienced by youth in refugee camps and settlements may further develop understanding of adverse psychosocial outcomes. The present study focused on violence as the primary adversity for clarity and parsimony, and to address gaps in the evidence-base.

## Conclusion

Analysis has indicated that refugees living in camps in LMICs face specific health risks and distinct patterns of morbidity and mortality compared with internally displaced persons and refugees in high-income settings (Spiegel *et al*. 2010). Intervention design and service delivery needs to take into account these specificities, taking into account ongoing insecurity, limitations in access to basic needs, and stressors at the household level, which may expose children to ongoing violence. The risk and protective factors for adverse mental health and psychosocial outcomes of children living in refugee camps in LMICs may differ than those populations on whom the majority of evidence exists, however the current evidence-base limits the ability of program planners, policy makers and donors to adequately address these differences. The findings from the present study indicate a need to combine rigorous research on prevalence and predictors of violence in refugee camps and settlements with assessment of mental health outcomes.

## Supplementary material

For supplementary material accompanying this paper visit http://dx.doi.org/10.1017/gmh.2017.17. click here to view supplementary material
