# Supplementary material for: Latent class analysis of violence against adolescents and psychosocial outcomes in refugee settings in Uganda and Rwanda
Source: Glob Ment Health (Camb). 2017 Oct 16;4:e19. doi: 10.1017/gmh.2017.17 (PMC5719474; doi:10.1017/gmh.2017.17)
Supplement: Supplementary file 1 [file S2054425117000176sup001.docx]

**Supplementary file 1:**

**Latent class fit statistics:**

Uganda

| Number of Classes | BIC | VLMR LR test p-value | LMR LR test p-value |
| --- | --- | --- | --- |
| 1 | 5471.417 |  |  |
| 2 | 3649.318 | < 0.0001 | < 0.0001 |
| 3 | 3441.116 | < 0.0001 | < 0.0001 |
| 4 | 3447.432 | 0.0911 | 0.0930 |

Rwanda

| Number of Classes | BIC | VLMR LR test p-value | LMR LR test p-value |
| --- | --- | --- | --- |
| 1 | 1969.714 |  |  |
| 2 | 1676.567 | < 0.0001 | < 0.0001 |
| 3 | 1686.956 | 0.0007 | 0.0008 |
| 4 | 1738.034 | 0.5501 | 0.5552 |
